# Mini-subvastus approach for total knee arthroplasty in obese patients

**DOI:** 10.4103/0019-5413.65157

**Published:** 2010

**Authors:** Nilen Shah, Nilesh G, Narendra Patel

**Affiliations:** Consultant Orthopaedic Surgeon, Bombay Hospital, Mumbai, India

**Keywords:** Mini-subvastus approach, total knee arthroplasty, obesity

## Abstract

**Background:**

Mini-subvastus approach for Total Knee Arthropalsty allows a faster recovery. It is traditionally not utilized for obese patients because of difficulty in exposure of the knee and eversion of the patella. We hypothesized that obesity should not really cause a problem for patients undergoing a TKA with the mini-subvastus approach as the anatomy of the quadriceps in the obese and the nonobese patient population is the same. We present an analysis of the use of mini-subvastus approach in obese patients.

**Materials and Methods:**

97 obese patients (109 knees) 81 females + 16 males with mean age 64 years underwent total knee arthroplasty (TKA) by mini-subvastus approach between January 2006 to July 2007. 16 patients (18 kness) were morbidly obese. All patients were prospectively evaluated by pre- and postoperative Knee Society and function score. The average follow-up was 18 months (range from 1 to 3 years) with minimum 1 year follow-up.

**Results:**

The approach provided adequate exposure in all knees, with an average surgical time of 90 minutes. The patella could be everted easily after the tibial and femoral cuts. The average Knee Society score improved from 42 to 89 and the function score from 48 to 65. The complications included medial collateral ligament injury (one case) and patellar tendon avulsion (one case).

**Conclusion:**

Our results compare favorably with other reported series in obese patients. The mini-subvastus approach can be considered in obese patients.

## INTRODUCTION

Obesity has been reported to be associated with degenerative osteoarthritis (OA) of the knee joint^1^,^2^ due to increased joint load. The body mass index (BMI), also called Quetelet’s index, relates well with the body fat percentage and is the preferred method for assessment of potential health risks associated with excess body weight.^1^ The index is derived by dividing an individual’s weight (in kilograms) by his or her height (in meters) squared. The World Health Organization (WHO) has devised a classification system for overweight adults according to BMI: normal = 18.5-24.9, overweight = 25-29.9, obese = 30-39.9, and morbidly obese = ≥40. There is a general consensus that excessive weight is a risk factor for total knee arthroplasty (TKA) as obese patients have a higher incidence of intra-operative and post-operative complications.^3^–^5^ such as wound healing problems, infections, higher incidence of damage to medial collateral or patellar tendon ligament, lower Knee society scores and higher revision rates due to aseptic looseneing.

TKA in obese and morbidly obese patients is performed through a standard medial parapateller arthrotomy, and the overall results in this subgroup of patients are inferior to that in nonobese patients.^3^,^4^,^5^ The subvastus approach, first described by Hoffmann in 1991,^6^ has the advantages of preserving extensor integrity and patellar blood supply, allowing better patellar tracking^7^,^8^ having less requirement for lateral release, and permitting early postoperative rehabilitation.^7^,^9^ The subvastus approach is considered to be relatively contraindicated in obese patients undergoing TKA^10^,^11^ although no clear reasons have been put forward for this. The major criticisms of this approach have been the poor and unpredictable exposure of the knee and difficulty with eversion of the patella.^11^,^12^ A recent article^12^ focused on the thigh girth of obese patients opined that if thigh girth was >55 cm, the subvastus approach should not be utilized as it is difficult to evert the patella in such patients.

The mini-subvastus approach has a smaller skin incision and the knee is approached through a quadriceps sparing technique, knee flexion is adjusted to expose different areas of the knee, retractors are used symbiotically and a mobile window is utilized with downsized instrumentation. Patellar eversion and tibiofemoral dislocation prior to bony cuts are also avoided.^13^ Thus it not only has smaller incision but also reported to have an earlier recovery, without compromising on the eventual goals of proper component positioning and alignment.

We hypothesized that obesity should not really cause a problem for patients undergoing a TKA with the mini-subvastus approach as the anatomy of the quadriceps in the obese and the nonobese patient population is the same. We are presenting an analysis of the use of mini-subvastus approach in obese patients.

## MATERIALS AND METHODS

All obese (BMI>30) patients with the diagnosis of primary OA, who underwent TKA with the mini-subvastus approach between January 2006 to July 2007 were included in the study. None of these patients had had any previous knee surgery. There were 81 females and 16 males with mean age 64 years (ranged 49-80 years). Twelve patients had staged bilateral knee arthroplasty. 16 patients (18 knees) were morbidly obese (BMI >40). Thigh girth was measured in centimeters, 15 cm above the adductor tubercle.^12^ The thigh girth in the obese group (n-81, 91 knees) was average 50.17 cm (range 45-58 cm). The thigh girth in the morbidly obese group (n-16, 18 knees) was average 61.01 cm (range 55-72 cm). There were 46 knees with a thigh girth above 55 cm. Patients were included in this study irrespective of degree of varus/valgus deformity or degree of flexion contracture and range of motion. There were 4 knees that were stiff with a flexion arc of less than 50 degrees.

After preoperative hematological tests, urine test, an ECG, Echo-cardiogram and a chest X-ray the knees were operated through a mini-subvastus approach.

### Operative procedure

An upper thigh tourniquet (pressure 100 mm Hg above the systolic pressure) was used in 15 knees. The rest of the knees were operated without a tourniquet. In cases, where a tourniquet was not used, tranexamic acid^14^ in a dose of 15 mg/kg was given 30 minutes prior to surgery and two doses of 10 mg/kg were given 3 hours and 6 hours post surgery. Intra-operatively saline-adrenaline (1:300000) was infiltrated into the skin and subcutaneous tissue to reduce bleeding. In cases where a tourniquet was utilized, it was released prior to closure after cementation of the final components.

A skin incision was made slightly medial to the midline of the knee, extending from the superior pole of the patella to the tibial tubercle, with the knee in 90° of flexion. Dissection was carried out until the extensor apparatus was exposed. A medial flap was raised to identify the inferior margin of the vastus medialis. The vastus medialis was bluntly dissected off the intermuscular septum. An ‘L’ shaped capsulotomy was made with the horizontal limb of the L along the inferior margin of the vastus medialis up to the superior pole of the patella. The vertical limb of the L extended from here up to the tibial tubercle. At this stage it was possible to displace the patella laterally to expose the suprapatellar synovium, which was then divided medially keeping the suprapatellar pouch intact. This method allows patella to be subluxed in the lateral gutter further. If there were prominent osteophytes in the trochlear region, these were removed with the knee in extension. A Hohmann retractor was placed, retracting the subluxated patella. A preliminary soft tissue release was carried out from the medial tibia until the mid-coronal plane in the varus knees. The knee was then flexed, with the Hohmann retractor retracting the patella in the lateral gutter. This flexion of the knee with the patella retracted laterally could be easily achieved when the knee had a good range of movement. When the knee was stiff this manoeuver could be difficult. In these cases, (stiff knees) quadriceps muscle needed to be dissected off the medial intemuscular septum more proximally. Also all hypertrophic osteophytes from the trochlear region and from the patella required meticulous removal so that the patella could be displaced laterally. Once the patella was displaced laterally satisfactorily the knee was gently flexed with a Hohmann retractor placed laterally to keep the patella laterally subluxed. Now another retractor was placed medially around the medial femoral condyle to expose the knee. The cruciate ligaments were excised and this allowed the knee to be flexed a little more. Care was taken to ensure that the patellar tendon insertion remained intact.

Overhanging osteophytes were removed from the femur and the tibia. The distal femoral cut was made first using a downsized intramedullary jig. Remnants of the cruciate ligaments were further excised. (Only Posterior stabilized implants were utilized) The knee was then extended. The lateral tibial plateau was exposed in extension by sharp dissection, taking care to avoid injury to the patellar ligament. The fat pad was not excised. A retractor was placed around the lateral tibial metaphysis and another was placed around the medial tibial metaphysis. The knee was flexed again and the third Hohmann retractor was used posteriorly to subluxate the tibia forward. An extramedullary jig was utilized to cut the proximal tibia at an adequate depth and angle. In some cases, the cut tibial bone was removed in piecemeal. The knee was now extended and the menisci were excised in extension. Care was taken whilst removing the medial meniscus, not to damage the medial collateral ligament. Overhanging osteophytes were removed from the posteromedial tibia if present. A spacer block was utilized to check the extension space. If necessary, further medial or lateral release was done to establish a proper extension space and to check the alignment. Thereafter, the femur was sized and and the appropriate AP cutting jig was placed on the femur such that the flexion space equaled the extension space. Due care was taken to avoid notching of the anterior cortex of the femur by the anterior cut. Champher and the notch cuts were made next. In case of the high-flex (n=90) variety of knee prosthesis, the posterior femur was recut appropriately. The highflex implant was used whenever the knee which was operated had an excellent pre-operative ROM i.e. > 120 degrees)

The patella was everted only after the femoral and tibial cuts had been made. Hypertrophic suprapatellar synovium and overhanging osteophytes from the patella were easily removed after everting the patella. The jig was utilized to size and resect the patella if patellar resurfacing was to be carried out (n=3). The patellar resurfacing was carried out when there were prominent arthritic trochlear and patellar lesions. Trial components were inserted and a careful check was made regarding the range of movement, stability, and patellar tracking. If posterior femoral osteophytes were present, they were removed using a curved osteotome. If required, posterior capsular release was carried out. The bony surfaces were washed with pulsatile lavage, dried, and the appropriate components were cemented and the trial insert was placed into the tray. The knee was brought to full extension to pressurize the bone-cement interface during polymerization. After the cement had cured, the trial insert was removed and the entire periphery of both the femoral and tibial implants was checked for any extruded cement, which was removed if present. The definitive tibial insert was placed after adequately cleaning and drying the tibial implant. Thorough lavage was given.

An apical stitch at the angle of the ‘L’ was first taken to ensure that the capsule was neither advanced nor recessed. The rest of the closure was routine. The knee was infiltrated with 20 ml of mixture containing 0.25% bupivacaine, cefuroxime, and normal saline. A bulky dressing was applied for the first 24 hours. A femoral nerve catheter was inserted with the help of a nerve stimulator (Stimuplex, Braun) and 10 ml of a mixture containing 2% lignocaine and 0.25% bupivacaine was injected at 4-hourly intervals for 1 day. For 24-48 hours postoperatively a cryocuff was utilized on the operated knee.

The knee implants utilized included Zimmer NexGen Legacy PS in 18 knees, Zimmer NexGen Highflex in 39 knees, Zimmer NexGen Gender solution in 51 knees and PFC Sigma (Cruciate substituting) in 1 knee to give a total of 109 knees.

Static quadriceps exercises and straight leg raising(SLR)exercises were started from day 0 and range of motion (ROM) exercises beginning from day 1. Below-knee thrombo-embolism stockings for both lower limbs were utilized. Chemical prophylaxis for DVT was not given. Patients were encouraged to get out of bed and walk as tolerated from day 1. The patients were discharged when they were able to walk to the bathroom, sit on a chair and were on oral painkillers. They were encouraged to exercise at home to maintain and improve the ROM of the knee.

The knees were evaluated pre- and postoperatively by the American Knee Society clinical and functional score.^15^ Standard anteroposterior and lateral view radiographs were obtained in the recovery room [Figure 1A]. At 6 weeks an additional merchant view (skyline view) radiograph was taken. The postoperative follow-up was at 6 weeks, 3 months, 6 months, and yearly thereafter. The final evaluation has been done at a minimum of 1 year follow-up with an average follow-up of 18 months (Range from 1 to 3 years) [Figure 1B].

**Figure 1A:**
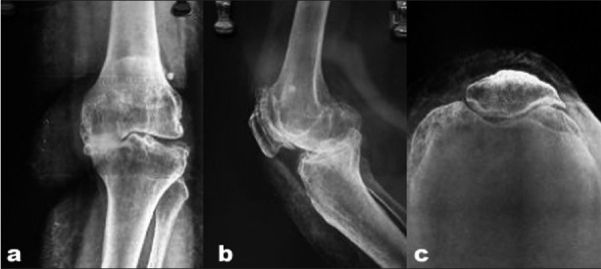
Pre-operative anteroposterior (a) lateral (b) and skyline (c) views of an obese patient showing advanced knee osteoarthritis

**Figure 1B:**
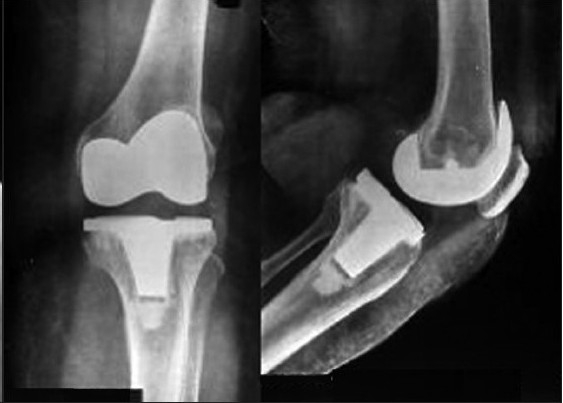
Anteroposterior and lateral radiographs at 18 month followup post mini-subvastus TKR of the same patient

## RESULTS

The mini-subvastus approach provided satisfactory exposure in all knees that were operated. In no case was this approach abandoned. The approach proved equally effective in the knees that were expected to be difficult, i.e., morbidly obese patients, with large thigh girth, (above 55 cm) irrespective of any degree of varus or valgus deformity [Figure 2A, 2B‐a]. The average surgical time was 90 minutes (range: 60-120 minutes). The average blood loss was 400 cc (range: 200-800 cc). None of the knees required a lateral release. The patellar tracking was immaculate in every case and in fact it was difficult to displace the patella laterally after 30° of knee flexion. In our study, the average incision length was 10 cm [Figure 2B, b].

**Figure 2A:**
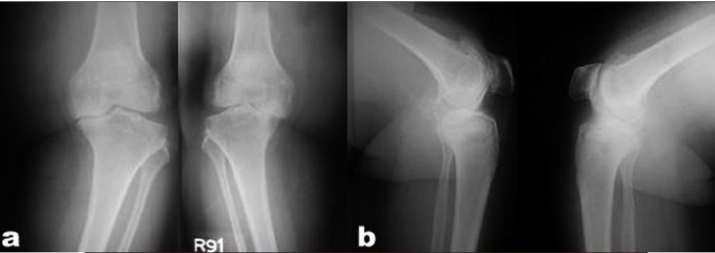
Bilateral anteroposterior (a) and lateral (b) knee radiographs of an obese patient with advanced knee osteoarthritis

**Figure 2B:**
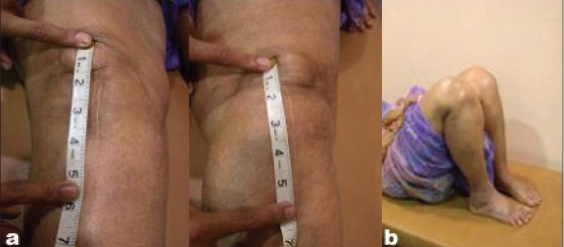
(a) Clinical photograph bilateral incision lengths of 4 inches (b) Post-operative bilateral full knee flexion at 8 weeks post surgery

The Knee Society score improved from the preoperative average of 42 (range: 17-62) to 89 (range: 72-95) postoperatively. The Knee Society functional score improved from the preoperative average of 48 (range: 15-60) to 65 (range 50-80) postoperatively. The quadriceps recovery was very early and 95% of the patients (103 knees in 87 patients) were able to do an active SLR by day 2. 85% of the patients (77 out of 91 patients) were able to walk unaided with a reciprocal gait by day 4. Only 5% of the patients (4 patients with 6 knees) had a 10° quadriceps lag after day 4, and this was seen in those knees that had a greater than 10° flexion deformity.

89/109 knees had excellent Range of movement of the knee with a flexion arc equal or greater than 120 degrees at last follow-up. 20/109 knees had a range of movement from 90 to 120 degrees at last follow up. Overall, the mechanical alignment of the lower limb was satisfactory, with an average valgus angle of 6° (range: 3°-9°). The component positioning and cementation appeared satisfactory [Figure 2B and C]. There were two knees with retained posterior cement.

**Figure 2C:**
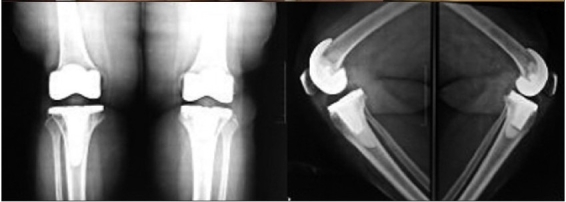
Bilateral anteroposterior and lateral radiographs at 18 month, follow-up of the same patient

There was one case of partial medial ligament injury. This injury was recognized on the table and end-to-end suture was possible. The knee was stabilized well in a hinged knee brace, which was given for 6 weeks postoperatively.^16^ The knee has remained stable at 18 month follow-up. There was one case of patellar tendon avulsion from the tibial tubercle. This female patient was morbidly obese and had had a successful contralateral total knee replacement 6 months earlier. The knee, with the patellar tendon avulsion, had a preoperative range of motion of 20°-80°. The surgical procedure appeared routine but for a partial avulsion of the patellar tendon from the tibial tubercle. This was recognized but was believed to be minor and the knee was protected in a long leg brace. On the third postoperative day, while the patient was walking, there was total avulsion of the patellar tendon from the tibial tubercle. She was re-explored and the tendon was reattached with suture anchors. It is now 12 months since the second surgery and the patient is able to walk without support but has an extensor lag of 5° with flexion from 0° to 100°. No wound complications and infection were noted. There was no clinical evidence of DVT in any of our patients.

**Table 1:** Patient demographics

	Obese group	Morbidly obese group
Total no. of patients (*n*=97)	81	16
Total no. knees (*n*=109)	91	18
Varus knees		
Mild deformity (<15°)	79	12
Severe deformity (>15°)	10	5
Valgus knees	2	1
Average height	153.75	149.69
Average weight	75.36	97.30
Average BMI	33.52	43.29
Average thigh girth	50.17	61.01
Gender		
Male	16	0
Female	65	16

## DISCUSSION

The mini-subvastus approach for TKA preserves quadriceps integrity, has a lesser incidence of lateral retinacular release^7^ and promotes an earlier recovery as compared to the medial parapatellar approach.^9^ However, the mini-subvastus approach is considered as a contraindication in obese patients because of the difficulty in exposure of the knee and eversion of the patella 11,12 Young *et al*. considered a large thigh girth (>55 cm) as a contraindication to the mini-subvastus approach because of the difficulty in eversion of the patella.^12^ We believed that the exposure would not be difficult as the anatomy of the quadriceps in the obese and the non-obese patients is the same and the eversion of the patella although difficult initially during the approach would not be so diffcult after the osteotomy of the femur and the tibia as this would have effectively relaxed the quadriceps muscle.

We found that the patella can be displaced laterally easily if the vastus medialis is released adequately from the intermuscular septum. We released as much of the muscle as was necessary to displace the patella easily. Synovial division also helped in the lateral displacement of the patella. The synovium was divided medially without violating the suprapatellar pouch. It was possible to flex the knee more (if the knee was stiff earlier) by the release of the vastus medialis from the septum and the removal of all osteophytes. Release of the vastus medialis from the septum may be considered akin to a limited quadricepsplasty.

We did not evert the patella primarily at all but only displaced it laterally. The offending tibiofemoral osteophytes were removed, keeping the patella located in the trochlear groove in varus knees. Overhanging medial patellar osteophytes, if present, can be removed with the knee extended and by just tilting the patella. In valgus knees, the osteophytes from the lateral femoral surface were removed with the patella subluxed in the lateral gutter. (Lateral tibial osteophytes if present were most easily removed after tibial cut and after fully flexing the knee.) Removal of the osteophytes and soft tissue release ‘debulked’ the knee and the exposure became progressively easier. Patellar eversion for resurfacing or debulking (removal of overhanging superior and lateral osteophytes) can be done relatively easily after the bony cuts of the tibia and femur have been made, as the quadriceps gets relaxed.

Performing the surgery through a smaller incision needs repositioning of the knee in varying degrees of extension and flexion and although all the parts of the knee may not be seen simultaneously, adequate exposure is obtained to perform each step of the operation. Symbiotic use of retractors and the use of a mobile skin window to accomplish the sequential steps of the operation are the key factors in the mini-subvastus approach.^13^

There has been some concern over the issue of component malalignment with minimally invasive TKA,^17^ especially in obese patients. In our series we did not observe any component malalignment, which has also been the case with other series.^18^,^19^ However, a detailed analysis of component positioning would form the basis of another study. We believe that component malalignment can be avoided by careful identification of the anatomical landmarks and the use of standard surgical jigs. When the lateral tibial plateau is adequately exposed, the risk of tibial malalignment or downsizing can be minimized. Similarly, the transepicondylar axis can be drawn and internal rotation of the femoral component can be avoided. The extensor mechanism is not violated, in the subvastus approach which provides inherent stability to the patellofemoral joint. Minimally invasive subvastus TKA should be attempted only after considerable surgical experience is gained in placing the components through a standard approach.

Only three patellae were resurfaced (3/109); the remaining were not resurfaced and hence the likelihood of over-stuffing the patellofemoral joint was low. The patellofemoral tracking in each and every knee was good and there was no lateral tilting of the patella. In the medial parapatellar approach, judging the patellofemoral tracking can be difficult as the medial supporters of the patella have been divided^7^,^8^ whereas in the subvastus approach judging the tracking of the patellofmoral joint is much easier as the medial supporters of the patella remain intact. There is a higher incidence of lateral releases in knees operated by the medial parapatellar approach.^7^,^20^ This increases the risk of a ‘cold patella,’ as vascularity of the patella can be jeopardized.^6^,^20^ The subvastus approach is less invasive than the standard approach as far as the vascular and muscular anatomy of the quadriceps is considered. The blood supply to patella is maintained via the intramuscular descending genicular artery. In the subvastus approach as the extensor mechanism is not violated, patellofemoral tracking remains immaculate and can be readily visualized, thus obviating the need for unnecessary lateral releases.^7^,^20^

The overall rate of complications in obese and morbidly obese patients is high even with the parapatellar approach.^3^,^5^,^21^ Winarsky *et al*. observed 4 cases of medial collateral ligament avulsion, 1 case of patellar tendon avulsion, and 11 cases (22%) with wound complications; in addition, 5 cases (10%) had a deep joint infection in their series of 40 morbidly obese patients.^5^ Amin *et al*. observed 7 cases with superficial wound complication, 2 cases of deep joint infection, and 4 cases of deep vein thrombosis in their series of 41 morbidly obese patients.^3^ In our series, there was one case each (0.91%) with partial medial collateral ligament injury and of patellar tendon avulsion from the tibial tubercle.The incidence of 0.21-8%,^22^,^23^ of extensor mechanism disruption in TKA even in non-obese patients operated with a standard parapatellar approach has been described.

Operating on larger knees (thigh girth >55 cm) was more challenging, but it was not associated with increased complications or a worse outcome; it does not seem to be a contraindication to the mini-subvastus approach as we had good results in 45 out of 46 knees with thigh girth >55 cm. Skin necrosis can be avoided by careful subfascial dissection and by not undermining the lateral flap. As is well known, the vascularity of the knee is lateral based and by keeping the lateral flap thick, skin necrosis can be avoided.

It is our observation that the mini-subvastus approach is more difficult to perform in patients having a limited range of knee movement; i.e. a stiff knee. (Stiff knee is defined as knee with < 50° range of movement)^24^ A stiff knee has intra- articular and periarticular adhesions restricting motion, Additionally there may be a contracture of the quadriceps^25^ which also would impair flexion. Large osteophytes in the femoral trochlea and the patella would act as mechanical blocks to flexion. Intra-operatively it was observed that it was necessary to remove all these osteophytes before the knee could be flexed safely. Release of the vastus medialis from the medial intermuscular septum mobilized the quadriceps muscle and allowed the patella to be subluxed in the lateral gutter. The more stiiff the knee was, the greater was the difficulty in subluxing the patella in the lateral gutter. Forcible attempts at flexion of the knee without subluxing the patella laterally could be a factor in injury to the patellar tendon and the medial collateral ligament.^16^,^22^,^23^ The case of patellar tendon avulsion in our series occurred in a morbidly obese lady with a stiff knee. On the other hand, a knee with a good range of movement indicates pliability of the quadriceps mechanism, which can be easily exploited to slide the mechanism laterally for a successful mini-subvastus TKA. Asian patients, who form our patient base, generally have a well-preserved ROM despite advanced arthritis of the knee. However, what is the minimum preoperative Range of movement of the knee that would allow successful performance of TKA with the mini-subvastus approach is probably not known at present.

The total measured blood loss was 400 ml (range: 200-800 ml). This compares favorably with other reported series.^26^ As this approach involves releasing the vastus medialis from the intermuscular septum, we believe that it should be feasible in most routine primary cases needing a TKA and probably all cases where medial parapatellar arthrotomy would suffice. The mini-subvastus approach for a TKA may not be suitable for those cases where an extension of the medial parapatellar approach is warranted, such as a quadriceps snip or tibial tubercle osteotomy. The drawback of our study is that we do not have a control group and we are comparing our results with historical controls.

To conclude, the mini-subvastus approach offers adequate intra-operative exposure even in obese and morbidly obese patients. It did not result in increased complications in our hands, even in morbidly obese patients with relatively greater thigh girth.
